# Early onset of stomatal closure confounds current interpretations and applications of iso‐/anisohydry theory

**DOI:** 10.1111/nph.70847

**Published:** 2025-12-30

**Authors:** Matthias Arend, Richard L. Peters, Cedric Zahnd, Mladen Ognjenovic, Günter Hoch, Ansgar Kahmen

**Affiliations:** ^1^ Physiological Plant Ecology, Department of Environmental Sciences University of Basel Schönbeinstrasse 6 CH‐4056 Basel Switzerland; ^2^ Plant Ecology, Department of Environmental Sciences University Trier Behringstraße 21 Trier 54296 Germany

**Keywords:** drought response strategy, hydroscape area, iso‐/anisohydry, stomatal regulation, water potential

## Disclaimer

The New Phytologist Foundation remains neutral with regard to jurisdictional claims in maps and in any institutional affiliations.

We critically assessed the iso‐/anisohydry concept, which builds on the assumption of tight stomatal regulation of water potential during tree dehydration. We found a consistent pattern of early stomatal closure in mature trees of eight tested species that precedes the decline of water potential in advanced stages of tree dehydration. This calls for a revision of the iso‐/anisohydry concept in which early stomatal closure prevents trees from entering an advanced stage of dehydration.

Global observations of drought‐induced tree mortality have encouraged intense research on plant hydraulic traits and derived metrics that can be used to quantify a tree's drought response strategy and define functional stress limits (Hartmann *et al*., 2018; Walthert *et al*., 2021; McDowell *et al*., 2022). Besides the discovery of various thresholds of plant hydraulic integrity (e.g. percentage loss of hydraulic conductance), the more than 80‐year‐old iso‐/anisohydry theory (Walter, 1931) has received new attention. Essentially, it builds on a plant's dilemma of absorbing CO_2_ through opening its stomata, thereby running the risk of excessive water loss through transpiration. Based on this, plants have been divided into iso‐ or anisohydric drought response strategy types (e.g. Leuschner *et al*., 2021 and references herein). Broadly, isohydric trees adopt a conservative water use strategy at the expense of carbon gain due to strict stomatal regulation during hydraulic stress. Anisohydric trees, on the other hand, favour CO_2_‐uptake with the risk of excessive water loss and hydraulic failure. As a result, isohydric trees target less negative water potentials (Ψ) than anisohydric trees and are therefore considered to be more resistant to drought (McDowell *et al*., 2008). In recent years, however, divergent definitions and terminology of isohydrocity have emerged (Martinez‐Vilalta & Garcia‐Forner, 2017; Ratzmann *et al*., 2019), leading to a variety of coexisting interpretations and thus complicating practical applications in comparative tree ecology (Hochberg *et al*., 2018; Leuschner *et al*., 2021).

The concept of iso‐/anisohydry is based on the popular assumption that gradual stomatal adjustments mediate a species‐specific trade‐off between transpirational water loss and CO_2_‐uptake on a tree's path to dehydration (Tardieu & Simonneau, 1998; McDowell *et al*., 2008). This process is orchestrated by a multitude of chemical and hydraulic signals, acting on different levels of plant organization and environmental interaction on stomatal aperture and thus leaf transpiration and CO_2_‐uptake (Comstock, 2002). It is further assumed that tight coordination of stomatal conductance (*g*_s_) and Ψ is the critical component in this regulatory network (Joshi *et al*., 2022), although the stringency of this interaction was recently questioned (Martinez‐Vilalta & Garcia‐Forner, 2017). Based on the role of stomata in regulating transpiration, species‐specific *g*_s_ vs Ψ relationships were constructed, which eventually led to the current theory of a continuum rather than a dichotomy of iso‐/anisohydric behaviour across species (Klein, 2014). This work was an attempt to quantitatively describe the iso‐/anisohydric behaviour of trees and develop physiologically based criteria for assessing their drought response strategy (Peters *et al*., 2023). However, to make the iso‐/anisohydric concept operational, fully quantitative metrics for iso‐/anisohydric behaviour are needed.

**Fig. 1:**
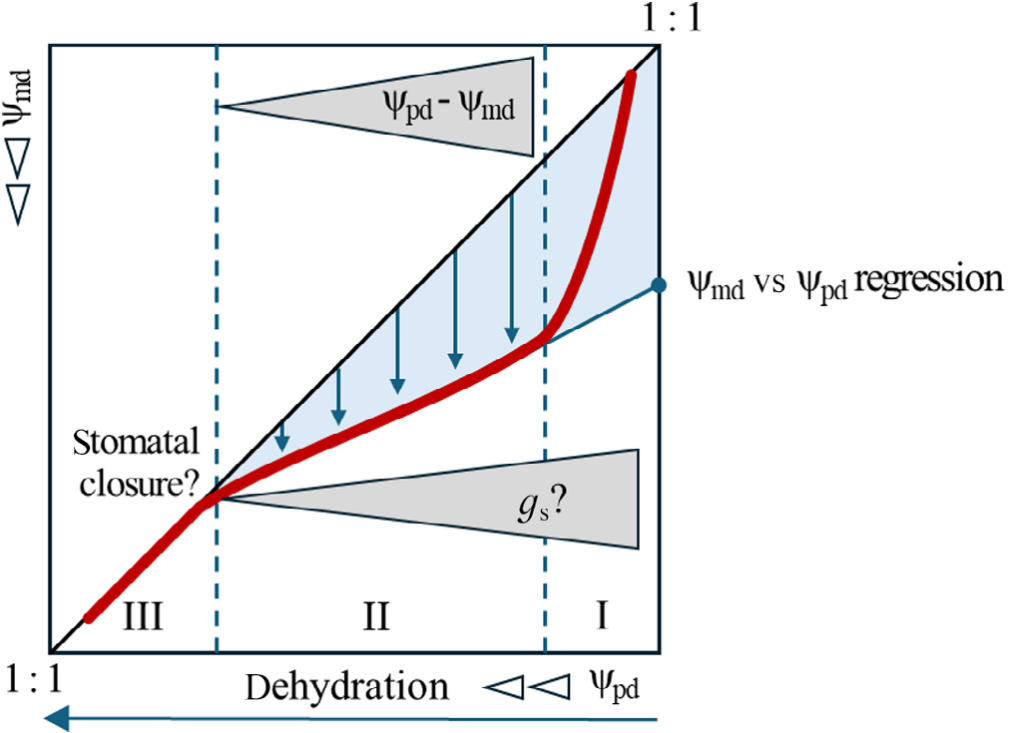
Conceptual presentation of the Ψ_pd_ vs Ψ_md_ dehydration trajectory based on current theory (Hartmann *et al*., 2021). The Ψ_pd_ vs Ψ_md_ trajectory (red bold line) displays three distinct phases of tree dehydration in a 1 : 1 plot projection (dehydration horizontal arrow; phase transitions vertical dashed lines). In Phase I, Ψ_md_ shows rapidly increasing deviations from Ψ_pd_, linearly disconnecting from the 1 : 1 line. This early phase of dehydration likely includes stomatal responses to fluctuations of incident light and atmospheric vapor pressure deficit. In Phase II of progressing dehydration, declining deviations of Ψ_md_ from Ψ_pd_ (vertical arrows) approach linearly the 1 : 1 line as a result of gradual stomatal regulation. In Phase III, Ψ_md_ does not further deviate from Ψ_pd_ as stomates are closed. The hydroscape area (HA) is the blue‐shaded area bound by the regression line, 1 : 1 line and the *y*‐axis of the Ψ_pd_ vs Ψ_md_ plot. Regulation of Ψ_md_ by *g*_s_ in Phases I and II is a fundamental assumption of current iso‐/anisohydry theory and critical for the interpretation of the HA metric as an integrative measure of stomatal regulation. However, the range over which Ψ_md_ is actually regulated by *g*_s_ and the point of stomatal closure remain to be empirically proven. Ψ_pd_, predawn tree water potential; Ψ_md_, midday tree water potential; *g*_s_, stomatal conductance.

Over the last decade, progress has been made in developing a conceptual framework that proposes new quantitative metrics of isohydrocity and thus drought resistance (Martinez‐Vilalta *et al*., 2014; Meinzer *et al*., 2016; Hartmann *et al*., 2021). It assumes that the extent to which minimum water potentials at midday (Ψ_md_) deviate from preceding predawn water potentials (Ψ_pd_) reflects stomatal regulation, with large deviations indicating low stomatal control. This raised the idea that the slope of the Ψ_pd_ vs Ψ_md_ regression is a useful metric to describe the operational range for stomatal regulation under progressing drought (Fig. 1). Furthermore, this concept assumes stomatal closure at the point where Ψ_md_ equals Ψ_pd_. Based on these assumptions, a ‘hydroscape area’ (HA) is defined to quantify the stringency of stomatal control over Ψ_md_. The advancement of the HA metric lies in the fact that it integrates all processes controlling Ψ_md_ over a large range of tree dehydration. Global analysis of Ψ_pd_ vs Ψ_md_ relationships has shown that HAs can separate species across a broad range of functional groups and biomes (Fu & Meinzer, 2019; Salvi *et al*., 2022). Despite these promising results, there are reasonable concerns about the applicability of the isohydrocity concept for assessing a tree's drought response strategy, as the fundamental assumptions of tight stomatal regulation of Ψ_md_ are not fully tested and studies on mature trees under natural droughts are largely lacking. This could explain the discrepancies in studies using different metrics of isohydrocity in different experimental setups to compare species‐specific drought response strategies (Martinez‐Vilalta & Garcia‐Forner, 2017; Fu & Meinzer, 2019; Li *et al*., 2019).

**Fig. 2:**
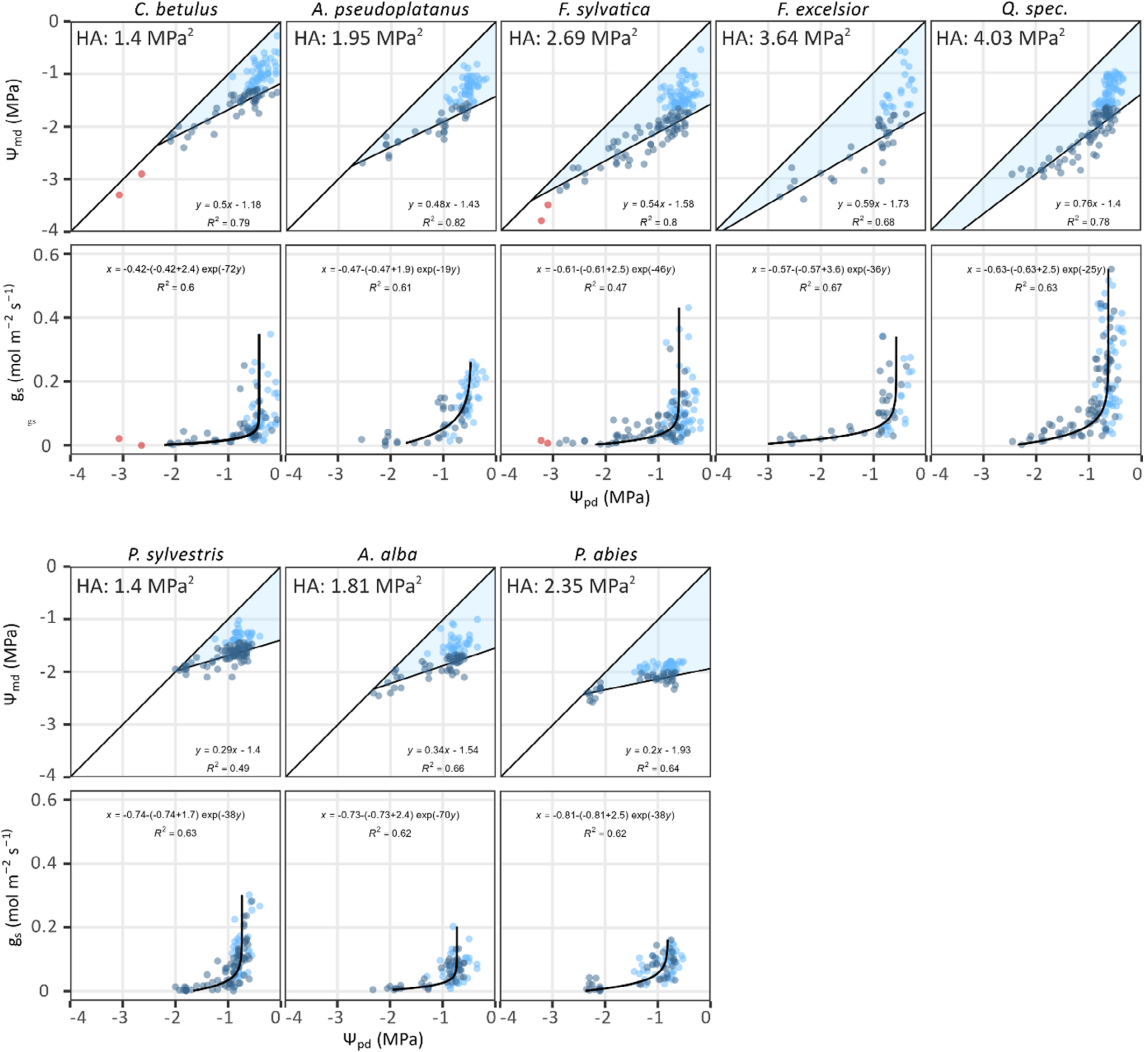
Relationships of Ψ_pd_ vs Ψ_md_ and vs *g*_s_ in the eight studied tree species (*Carpinus betulus*, *Acer pseudoplatanus*, *Fagus sylvatica*, *Fraxinus excelsior*, *Quercus spec.*, *Pinus sylvestris*, *Abies alba*, *Picea abies*). The obtained trajectories of Ψ_pd_ and Ψ_md_ reveal three phases of tree dehydration (see conceptual Fig. 1): (I) large, indefinite deviations of Ψ_md_ from Ψ_pd_ in the initial phase (Ψ_pd_ > −1.0 MPa), associated with large variations of *g*_s_, (II) almost linearly declining deviations of Ψ_md_ from Ψ_pd_ in the second phase, associated with *g*_s_ values dropping rapidly towards the instrumental detection limit, (III) low or negligible deviations of Ψ_md_ from Ψ_pd_ in the third phase (only reached in *Carpinus betulus* and *Fagus sylvatica*), associated with *g*_s_ values close to the instrumental detection limit. The hydroscape area (HA) in the Ψ_pd_ vs Ψ_md_ relationships is the blue‐shaded triangle bound by the 1 : 1 line, the *y*‐axis and the black linear regression line (inserts show the calculated HAs). The asymptotic regression models for the Ψ_pd_ vs *g*_s_ relationships are represented by black curves. Data points show all measurements that were collected in the analysed trees during the three‐years observation period (light‐blue‐shaded dotes: data first phase of tree dehydration excluded from linear regressions; grey dotes: data first and second phase of tree dehydration included in linear regressions to delineate HAs, orange dots: data third phase of tree dehydration excluded from linear regressions). Ψ_pd_, predawn tree water potential; Ψ_md_, midday tree water potential; *g*_s_, stomatal conductance; HA, hydroscape area.

Here, we use extensive observations of Ψ_pd_ and Ψ_md_ and midday measurements of *g*_s_ over 3 yr in five angiosperm and three coniferous species in a mature temperate forest at the Swiss‐Canopy‐Crane‐II site (Supporting Information Table S1; Peters *et al*., 2025a). The observation period covered the full spectrum of moisture conditions from wet to exceptionally dry (Table S2) in which trees came close to their previously reported thresholds of hydraulic integrity (Kahmen *et al*., 2022). Using these data, we constructed species‐specific relationships of Ψ_pd_ vs Ψ_md_ over a wide range of tree hydration (Fig. 2). In line with current theory (Fig. 1), the observed relationships reflect three phases of dehydration (Meinzer *et al*., 2016). Increasing deviations of Ψ_md_ from preceding Ψ_pd_, linearly disconnecting from the 1 : 1 line in the initial phase (I), declining deviations, linearly approaching the 1 : 1 line in the second phase (II) and small deviations following the 1 : 1 line in the third phase (III) (*cf*. *Carpinus betulus* and *Fagus sylvatica* in Fig. 2). Data from the second phase and data linearly extending into the first phase were selected to delineate HAs, assuming the slope of declining deviations of Ψ_md_ from Ψ_pd_ defines the operational range of stomatal control over Ψ_md_ under progressing drought (Table S3; the Materials and Methods section; Meinzer *et al*., 2016). In all species, the second phase started at a Ψ_pd_ of *c*. −1.0 MPa, while the endpoints, where Ψ_md_ becomes nearly equal to Ψ_pd_, varied. In the ring‐porous angiosperms (*Fraxinus excelsior*, *Quercus spec*.), we were confronted with some uncertainty in defining the second phase of dehydration and delineating HAs, as the endpoints were not reached during severe drought, and data showed strong scatter or no linearity. Random subsampling analyses confirmed this uncertainty but showed little effect on relative species comparisons of HAs (Notes S1; Fig. S1; Tables S3, S4).

Despite the uncertainty described above, we nevertheless calculated HAs to quantify drought response strategies for the investigated species (Fig. 2; Table S3). We were particularly interested in whether HAs were associated with wood functional classification or previously assessed ecological characteristics (Niinemets & Valladares, 2006). We obtained the largest HAs for the ring‐porous angiosperms *Quercus spec*. and *Fraxinus excelsior*. Conversely, the diffuse‐porous angiosperm *Carpinus betulus* and the conifer *Pinus sylvestris* showed the lowest HAs. Regarding ecological classification, the calculated HAs barely reflected the reported characteristic of the studied species. For example, the two species classified as the most drought‐tolerant species showed the highest (*Quercus spec*.) and lowest (*Pinus sylvestris*) HA of all species (Table S3); and *Fagus sylvatica* and *Picea abies*, which severely suffered and died during recent drought spells (Schuldt *et al*., 2020; Arend *et al*., 2021, 2022), both displayed HAs in an intermediate range. This inconsistent species ranking shows, in agreement with previous concerns (Li *et al*., 2019), that the drought tolerance classification of trees goes far beyond a description of their iso‐/anisohydric behaviour.

To gain deeper insights into the mechanisms that shape the obtained HAs and better explain the inconsistent species ranking, we tested in each species how the Ψ_pd_ vs Ψ_md_ trajectories related to stomatal regulation (Fig. 2). Similar to observations of Ψ_md_, we found large variations of *g*_s_ in the initial phase of tree dehydration, which likely reflect stomatal sensitivity to daily fluctuations of incident light and vapour pressure. During the transition to the second phase of tree dehydration, *g*_s_ dropped rapidly to low values and then remained at a level close to the instrumental detection limit. All eight species showed this early steep decline of *g*_s_, a mechanism that we have recently linked with growth regulation at the onset of drought (Peters *et al*., 2025a). To quantitatively describe these exponential relationships for each species separately, we employed asymptotic regressions and derived species‐specific metrics that define a critical point at which stomata begin to close in response to declining Ψ_pd_ (Table S3). This point of Ψ_pd_, after which *g*_s_ declined rapidly, ranged between −0.4 and −0.8 MPa across the studied species. Below a Ψ_pd_ of *c*. −1 MPa, *g*_s_ came close to the instrumental detection limit (which is within the range of previously detected stomatal closure; Peters *et al*., 2025a). Notably, the decline of *g*_s_ was very steep in some species (*Carpinus betulus, Abies alba*), while it was more gradual in others (*Acer pseudoplatanus*; Fig. 2). Regardless of these differences in stomatal sensitivity (quantified as *ρ*; Table S3), we show that stomata closed much earlier than the equalization of Ψ_pd_ and Ψ_md_ suggested by current iso‐/anisohydry theory.

With the finding described above, we failed to confirm the fundamental assumption of current iso‐/anisohydry theory that the Ψ_pd_ vs Ψ_md_ trajectory reflects the path to stomatal closure, which also contradicts the interpretation of HAs as an integrative measure of stomatal regulation. It is particularly remarkable that dynamic stomatal regulation of Ψ_md_ was limited in all species to the beginning of tree dehydration when trees are not yet experiencing severe hydraulic stress and risk of hydraulic failure. This seems to be counter‐intuitive, considering the function of stomata to regulate leaf transpiration and tree water status. However, similar observations have already been made in other stress‐physiological studies (Arend *et al*., 2013; Bréda *et al*., 1993; Li *et al*., 2019; Arend *et al*., 2021; Aranda *et al*., 2024), and most recently, we found that early stomatal closure coincides with drought‐induced growth cessation (Peters *et al*., 2025a). These observations of early stomatal closure during tree dehydration are scattered throughout the extensive plant‐hydraulic and stress‐physiological literature and thus received little attention in the current iso‐/anisohydry theory. This may explain why our results conflict with the definition of the HA metric as a measure of stomatal function under progressing drought.

If no further stomatal regulation is possible during tree dehydration, what other mechanisms shape the Ψ_pd_ vs Ψ_md_ trajectories after stomatal closure? And why does Ψ_md_ still deviate from Ψ_pd_? Here, nonstomatal controls of Ψ may become relevant, for example cuticular transpiration (Duursma *et al*., 2018; Wang *et al*., 2024) or capacitive water release from shrinking stems (Salomón *et al*., 2017; Peters *et al*., 2023) or embolized sap wood (Salleo *et al*., 2000; Hölttä *et al*., 2009). Although not actively regulated, their effect on Ψ is closely linked to daily fluctuations of atmospheric water demand, which makes it plausible that Ψ_md_ still deviates from Ψ_pd_ after stomatal closure. Given the large amount of water that trees save in their voluminous stems, capacitive water release from sapwood could play a particular role in buffering Ψ against rapid uncontrolled decline (Scholz *et al*., 2011). In this context, it is noteworthy that we obtained the largest HAs, and thus least stringent control of Ψ_md_, for the ring‐porous angiosperms (*Fraxinus excelsior*, *Quercus spec*.), which have lower sapwood proportions than the other studied species. This may underline the importance of capacitive water release to stabilise Ψ in advanced stages of tree dehydration. Thus, the variability of HA in different species may depend not only on their specific stomatal regulation but also on their ability to buffer water loss by nonstomatal controls. Quantifying these nonstomatal controls would be a next step to truly disentangle the processes that shape Ψ during tree dehydration. Continuous stem diameter readings, from which tree water deficits and estimates of Ψ can be extracted, could facilitate this research (Ehrenberger *et al*., 2012; Peters *et al*., 2025b).

Taken together, our observations provide physiological evidence for early onset of stomatal closure during tree dehydration. We found that stomata start to close rapidly when Ψ_pd_ falls below a value of −0.4 to −0.8 MPa. We therefore conclude that stomatal regulation serves to prevent the trees from entering an advanced stage of dehydration rather than to shape the further decline of Ψ afterwards. This contrasts sharply with current interpretations of iso‐/anisohydry that assume dynamic stomatal regulation across the full range of tree dehydration but supports the recent finding of low correlation between *g*_s_ and isohydrocity definitions (Martinez‐Vilalta & Garcia‐Forner, 2017). The limited range for dynamic stomatal regulation of Ψ calls for rethinking the physiological foundation of iso‐/anisohydry theory. Therefore, the HA metric derived thereof should be interpreted with care and not used as a sole index of stomatal control over Ψ during tree dehydration, at least when comparing temperate species. This may finally explain the still existing discrepancies in attempts – including our own – to utilize quantitative metrics of iso‐/anisohydry as measures of stomatal function and tree ecological classification. However, further studies on trees in other forest biomes and climate regions are required to derive a generally valid readjustment of iso‐/anisohydry theory.

## Materials and Methods

### Research site and study trees

All measurements were carried out at the Swiss‐Canopy‐Crane II (SCC II) research site in Hölstein/BL, Switzerland (47°26′17″N, 7°46′37″E; 550 m asl), which is situated in a mixed temperate forest of the Swiss Jura mountains. The site conditions are characterized by a calcareous loamy soil and a climate with 9.0°C annual temperature and 1009 mm annual precipitation. The technical infrastructure includes a canopy crane with a height of 45 m and a jib of 50 m. The upper tree layer of the site is dominated by adult individuals of European beech (*Fagus sylvatica* L.) and Norway spruce (*Picea abies* L.) with co‐occurring oak (*Quercus petraea* Liebl. x *robur* L.), hornbeam (*Carpinus betulus* L.), Sycamore maple (*Acer pseudoplatanus* L.), European ash (*Fraxinus excelsior* L.), Scots pine (*Pinus sylvestris* L.) and Silver fir (*Abies alba* Mill.).

### Study period and weather conditions

All measurements were carried out in the summer months (June to August) 2020 to 2022. This period included strongly contrasting weather conditions as the exceptionally warm and dry summer months in 2020 and 2022, as well as the rather cool and wet summer of 2021 (Table S2). Our study thus covered the full spectrum of moisture conditions and tree hydration. Weather data for this period and the corresponding long‐term trend (2003–2022) for comparison were collected from a nearby climate station operated by the Swiss meteorological service (station Rüneberg; 47°26′04″N, 7°52′45″E; 611 m asl).

### Measurement of predawn and midday water potential (Ψ_pd_ and Ψ_md_)

Water potentials were measured with a Scholander's type pressure chamber (PMS Instrument Company, Albany, OR, USA) on two excised twigs per tree, collected predawn (4:00 h to 6:00 h CET) and midday (12:00 h to 14:00 h CET) in the upper, sun‐exposed canopy of the study trees.

### Determination of hydroscape area

Hydroscape areas as metrics of isohydrocity were determined according to previously published work (Meinzer *et al*., 2016; Fu & Meinzer, 2019). In brief, Ψ_pd_ vs Ψ_md_ relationships were inspected visually to preselect a range of the second phase of the dehydration trajectory in which the deviation of Ψ_md_ from Ψ_pd_ dynamically declines with progressing drought. As statistical approaches were not applicable to the scattered and partly nonlinear data, we followed the following rules to define the final range for delineating the HA: the dry border of the second phase of the dehydration trajectory was set by the point where Ψ_pd_ became closest to or equal to Ψ_md_ under severe drought. It was taken as the starting point for running a set of linear regressions with an increasing number of data pairs towards the wet border of the Ψ_pd_ vs Ψ_md_ relationship where Ψ_md_ shows large deviations from Ψ_pd_. The regression yielding the highest R^2^ was used to describe a linear relationship between Ψ_pd_ and Ψ_md_. This best‐fit approach allowed us to find a regression line for delineating the HAs that covers the wet to dry range of the dehydration trajectory (first and second phase of the dehydration trajectory) but excludes data in the wet range that was likely influenced by fluctuations of incident light, temperature or atmospheric vapour pressure deficit (vertically oriented trend at the beginning of the dehydration trajectory). The area of the triangle bound by the regression line, 1 : 1 line and the *y*‐axis of the Ψ_pd_ vs Ψ_md_ plot was expressed as HA (MPa^2^). It was dependent on the slope of the obtained regression line, which was used as an additional test metric of isohydrocity (Table S3; Martinez‐Vilalta *et al*., 2014).

In the two ring‐porous species *Quercus spec*. and *Fraxinus excelsior*, the delineation of HAs by linear regression was subject to some uncertainty as the dry endpoint of the second phase of the Ψ_pd_ vs Ψ_md_ relationship was not reached or showed strong scatter. In *Quercus spec*., nonlinearity of the Ψ_pd_ vs Ψ_md_ relationship additionally compromised the employment of linear regression. We therefore conducted additional random subsampling analyses with the full data (except the few data points in Phase III of the Ψ_pd_ vs Ψ_md_ relationship; Notes S1; Fig. S1; Table S4) to test the uncertainty of the HAs and the resulting species ranking. We found low‐to‐moderate uncertainties in conifers and diffuse‐porous angiosperms, respectively, but high uncertainty in the two ring‐porous angiosperms *Fraxinus excelsior* and *Quercus spec*. However, the ranking of the HAs across the species was robust (Fig. S1; Tables S3, S4).

### Measurements of stomatal conductance (*g*_s_)

Instant leaf gas exchange was measured using a portable photosynthesis system (LI‐COR 6800, LI‐COR Inc., Lincoln, NE, USA) equipped with an illuminated leaf or needle chamber. All measurements were taken in the upper, sun‐exposed canopy at the same time when Ψ_md_ was measured (12:00 h to 14:00 h CET). The conditions inside the chamber were kept at 1000 μmol photon flux density and 500 μmol s^−1^ flow rate with ambient CO_2_, temperature and humidity. Variable needle mass inside the chamber was corrected by measurements of needle area using a bench‐top leaf area metre (LI 3100C–LI‐COR Inc., Lincoln, NE, USA).

### Regression modelling

To describe the relationship between Ψ_pd_ and *g*_s_, we applied the asymptotic regression model using the following parameterisation:

$$\Psi_{\mathrm{pd}}=a-\left(a-b\right)\times \exp\left(-\rho \times g_{s}\right)$$

where *a* is the maximum attainable value of Ψ_pd_, *b* is the initial value of Ψ_pd_ when *g*_s_ is 0, and *ρ* is the relative rate of increase of Ψ_pd_ when *g*_s_ increases. The vertical plateau of the fitted curve (*a*) represents the Ψ_pd_ at which *g*_s_ becomes less responsive to further increases in Ψ_pd_, which is reflected in the large variability of *g*_s_. When Ψ_pd_ drops below the value of *a*, stomatal regulation becomes more pronounced, with *g*_s_ decreasing exponentially. The coefficient *ρ* indicates the sensitivity of stomatal response, with higher values denoting a steeper decrease in *g*_s_ with decreasing Ψ_pd_. The range between a and b coefficients defines the modelled range of Ψ_pd_ values where *g*_s_ is most influenced by Ψ_pd_. Asymptomatic regression models were fitted in R using the nls() and NLS.asymReg() function. The validity of model fits was confirmed by appropriate diagnostic and graphical tools.

## Competing interests

None declared.

## Author contributions

MA, GH and AK designed the study. MA, GH, RLP and CZ collected the data in the field. MA, MO and CZ analysed the data. MA wrote the manuscript with contributions from all authors.

## Supporting information


**Fig. S1** Random subsampling analysis of hydroscape areas.
**Notes S1** Random subsampling analysis of hydroscape areas.
**Table S1** Measured tree water potential and stomatal conductance.
**Table S2** Weather conditions in the observation years 2020–2022.
**Table S3** Functional and ecological classification of the studied tree species compared to metrics of isohydrocity and stomatal regulation.
**Table S4** Results from the subsampling analysis averaged across all subsampling proportions.
Please note: Wiley is not responsible for the content or functionality of any Supporting Information supplied by the authors. Any queries (other than missing material) should be directed to the *New Phytologist* Central Office.

## Data Availability

All data are included in the article and Supporting Information (raw data; Table S1).
