# Supplementary material for: Early onset of stomatal closure confounds current interpretations and applications of iso‐/anisohydry theory
Source: New Phytol. 2025 Dec 30;249(6):2616–22. doi: 10.1111/nph.70847 (PMC12917450; doi:10.1111/nph.70847)
Supplement: Supplementary file 1 — Fig. S1 Random subsampling analysis of hydroscape areas. Notes S1 Random subsampling analysis of hydroscape areas. Table S1 Measured tree water potential and stomatal conductance. Table S2 Weather conditions in the observation years 2020–2022. Table S3 Functional and ecological classification of the studied tree species compared to metrics of isohydrocity and stomatal regulation. Table S4 Results from the subsampling analysis averaged across all subsampling proportions. Please note: Wiley is not responsible for the content or functionality of any Supporting Information supplied by the authors. Any queries (other than missing material) should be directed to the New Phytologist Central Office. [file NPH-249-2616-s001.pdf]

New Phytologist

***New Phytologist* Supporting Information**

Article title: Early onset of stomatal closure confounds current interpretations and applications of iso/anisohydry theory

Authors: Arend M, Peters RL, Zahnd C, Ognjenovic M, Hoch G, Kahmen A

Article acceptance date: 25 November 2025

The following Supporting Information is available for this article:

Figure S1 Random subsampling analysis of hydroscape areas (HAs).

Table S1 Measured tree water potential and stomatal conductance.

Table S2 Weather conditions in the observation years 2020 to 2022.

Table S3 Functional and ecological classification of the studied tree species compared to metrics of isohydrocity and stomatal regulation.

Table S4 Results from the subsampling analysis averaged across all subsampling proportions.

Notes S1 Random subsampling analysis of hydroscape areas (HAs).

Figure S1 Random subsampling analysis of hydroscape areas (HAs). Distributions of HAs calculated from random subsamples of different proportions (90, 70, 50, 40, 30 and 20%; dark to light grey) from the dataset selected for linear regression. Plots show the median (points), 50% interval (bold whiskers) and 95% interval (narrow whiskers). Green crosses are the HAs calculated from the full dataset. Note the changing scale of the y-axis.

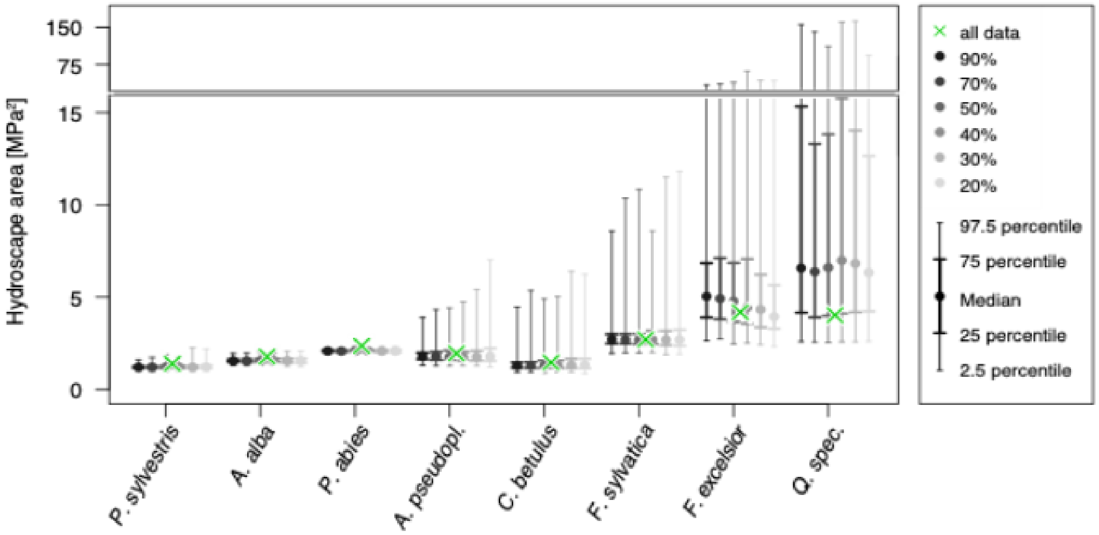

Table S1 Measured tree water potential and stomatal conductance. The values of predawn tree water potential (Ψ_{pd}), midday tree water potential (Ψ_{md}) and midday stomatal conductance (g_s) were collected in the summer half-years 2020 to 2022 (average of two measurements per tree).

species	Ψ_{pd} (MPa)	Ψ_{md} (MPa)	g_s (mol m^{-2} s^{-1})
A. alba	-0.35	-1.00	0.085
A. alba	-0.35	-1.53	0.071
A. alba	-0.50	-1.30	0.104
A. alba	-0.50	-1.38	0.164
A. alba	-0.50	-1.40	0.093
A. alba	-0.53	-1.48	0.041
A. alba	-0.55	-1.48	0.107
A. alba	-0.58	-1.45	0.103
A. alba	-0.60	-1.60	0.029
A. alba	-0.60	-1.70	0.044
A. alba	-0.60	-1.75	0.089
A. alba	-0.60	-2.10	0.088
A. alba	-0.65	-1.68	0.045
A. alba	-0.65	-1.70	0.066
A. alba	-0.68	-1.55	0.068
A. alba	-0.68	-1.70	0.084
A. alba	-0.68	-1.80	0.066
A. alba	-0.70	-1.35	0.025
A. alba	-0.70	-1.48	0.074
A. alba	-0.70	-1.73	0.134
A. alba	-0.70	-1.73	0.095
A. alba	-0.70	-1.80	0.083
A. alba	-0.73	-1.40	0.025
A. alba	-0.73	-1.68	0.102
A. alba	-0.73	-1.83	0.064
A. alba	-0.75	-1.50	0.032
A. alba	-0.75	-1.73	0.132
A. alba	-0.75	-1.73	0.095
A. alba	-0.75	-1.83	0.030
A. alba	-0.75	-2.00	0.084
A. alba	-0.78	-1.65	0.040
A. alba	-0.78	-1.65	0.050
A. alba	-0.78	-1.68	0.077
A. alba	-0.80	-1.10	0.203
A. alba	-0.80	-1.35	0.121
A. alba	-0.80	-1.53	0.057
A. alba	-0.80	-1.55	0.040
A. alba	-0.80	-1.58	0.077
A. alba	-0.80	-1.58	0.043
A. alba	-0.80	-1.80	0.095
A. alba	-0.80	-1.80	0.046
A. alba	-0.83	-1.08	0.133
A. alba	-0.83	-1.80	0.028
A. alba	-0.83	-2.05	0.046
A. alba	-0.85	-1.30	0.118
A. alba	-0.85	-1.40	0.008
A. alba	-0.85	-1.65	0.018
A. alba	-0.85	-1.55	0.081
A. alba	-0.85	-1.98	0.056
A. alba	-0.88	-1.50	0.078
A. alba	-0.88	-1.70	0.082
A. alba	-0.88	-1.75	0.065
A. alba	-0.88	-1.90	0.044
A. alba	-0.88	-1.95	0.101
A. alba	-0.90	-1.53	0.017
A. alba	-0.90	-1.70	0.093
A. alba	-0.93	-1.75	0.083
A. alba	-0.93	-1.98	0.029
A. alba	-1.00	-1.53	0.046
A. alba	-1.00	-1.63	0.062
A. alba	-1.03	-1.98	0.073
A. alba	-1.05	-1.18	0.096
A. alba	-1.25	-1.88	0.018
A. alba	-1.28	-2.00	0.010
A. alba	-1.28	-2.13	0.006
A. alba	-1.30	-1.65	0.046
A. alba	-1.35	-1.85	0.039
A. alba	-1.35	-1.95	0.015
A. alba	-1.40	-1.80	0.025
A. alba	-1.80	-2.10	0.009
A. alba	-1.80	-2.30	0.018
A. alba	-1.88	-2.10	0.008
A. alba	-1.90	-1.95	0.009
A. alba	-1.93	-2.00	0.005
A. alba	-1.93	-2.30	-0.001
A. alba	-2.00	-2.40	0.015
A. alba	-2.05	-2.20	0.009
A. alba	-2.23	-2.45	-0.002
A. alba	-2.33	-2.40	0.005
A. pseudoplatanus	-0.20	-1.10	0.213
A. pseudoplatanus	-0.25	-1.13	0.232
A. pseudoplatanus	-0.33	-1.10	0.148
A. pseudoplatanus	-0.35	-1.20	0.208
A. pseudoplatanus	-0.35	-1.25	0.152
A. pseudoplatanus	-0.38	-0.83	0.251
A. pseudoplatanus	-0.38	-1.05	0.192
A. pseudoplatanus	-0.38	-1.25	0.229
A. pseudoplatanus	-0.40	-1.15	0.244
A. pseudoplatanus	-0.43	-1.55	0.073
A. pseudoplatanus	-0.45	-1.05	NA
A. pseudoplatanus	-0.45	-1.18	0.212
A. pseudoplatanus	-0.45	-1.23	0.187
A. pseudoplatanus	-0.45	-1.53	0.263
A. pseudoplatanus	-0.48	-1.15	0.227
A. pseudoplatanus	-0.50	-1.05	NA
A. pseudoplatanus	-0.50	-1.15	NA
A. pseudoplatanus	-0.50	-1.20	0.127
A. pseudoplatanus	-0.50	-1.30	0.205
A. pseudoplatanus	-0.50	-1.35	0.151
A. pseudoplatanus	-0.50	-1.43	0.111
A. pseudoplatanus	-0.53	-1.25	0.228
A. pseudoplatanus	-0.53	-1.25	0.202
A. pseudoplatanus	-0.53	-1.33	0.121
A. pseudoplatanus	-0.53	-1.43	0.165
A. pseudoplatanus	-0.53	-1.55	0.093
A. pseudoplatanus	-0.53	-1.63	0.149
A. pseudoplatanus	-0.55	-0.90	0.180
A. pseudoplatanus	-0.55	-1.05	NA
A. pseudoplatanus	-0.55	-1.10	0.210
A. pseudoplatanus	-0.55	-1.20	0.129
A. pseudoplatanus	-0.55	-1.20	NA
A. pseudoplatanus	-0.55	-1.25	0.165
A. pseudoplatanus	-0.55	-1.25	0.099
A. pseudoplatanus	-0.55	-1.70	0.139
A. pseudoplatanus	-0.58	-1.10	0.161
A. pseudoplatanus	-0.58	-1.58	NA
A. pseudoplatanus	-0.58	-1.80	0.111
A. pseudoplatanus	-0.60	-1.33	0.109
A. pseudoplatanus	-0.60	-1.35	0.186
A. pseudoplatanus	-0.60	-1.40	0.128
A. pseudoplatanus	-0.60	-1.40	0.107
A. pseudoplatanus	-0.60	-1.85	0.064
A. pseudoplatanus	-0.63	-0.85	0.127
A. pseudoplatanus	-0.65	-1.73	0.120
A. pseudoplatanus	-0.65	-1.08	0.075
A. pseudoplatanus	-0.65	-1.25	0.199

species	Ψpd (MPa)	Ψmd (MPa)	g_s (mol m^{-2} s^{-1})
A. pseudoplatanus	-0.65	-1.63	0.111
A. pseudoplatanus	-0.65	-1.68	0.100
A. pseudoplatanus	-0.65	-1.68	0.080
A. pseudoplatanus	-0.68	-1.40	0.153
A. pseudoplatanus	-0.70	-1.53	0.156
A. pseudoplatanus	-0.70	-1.68	0.017
A. pseudoplatanus	-0.78	-1.55	0.120
A. pseudoplatanus	-0.78	-1.63	0.055
A. pseudoplatanus	-0.80	-1.30	0.031
A. pseudoplatanus	-0.85	-1.68	0.118
A. pseudoplatanus	-0.88	-1.55	0.015
A. pseudoplatanus	-0.90	-1.50	0.065
A. pseudoplatanus	-0.95	-1.45	0.035
A. pseudoplatanus	-0.98	-1.90	0.149
A. pseudoplatanus	-0.98	-2.00	0.065
A. pseudoplatanus	-1.00	-2.00	0.123
A. pseudoplatanus	-1.00	-2.10	0.116
A. pseudoplatanus	-1.00	-2.13	0.058
A. pseudoplatanus	-1.00	-2.25	0.036
A. pseudoplatanus	-1.05	-1.75	0.150
A. pseudoplatanus	-1.08	-1.90	0.023
A. pseudoplatanus	-1.10	-1.93	0.026
A. pseudoplatanus	-1.10	-2.15	0.136
A. pseudoplatanus	-1.30	-2.10	0.009
A. pseudoplatanus	-1.55	-2.10	0.008
A. pseudoplatanus	-1.88	-2.30	0.012
A. pseudoplatanus	-1.88	-2.30	0.008
A. pseudoplatanus	-2.05	-2.55	0.029
A. pseudoplatanus	-2.05	-2.60	0.042
A. pseudoplatanus	-2.10	-2.20	0.000
A. pseudoplatanus	-2.10	-2.35	0.010
A. pseudoplatanus	-2.10	-2.35	0.014
A. pseudoplatanus	-2.55	-2.70	0.020
C. betulus	-0.05	-0.6	0.150
C. betulus	-0.075	-0.275	0.164
C. betulus	-0.08	-1.35	0.081
C. betulus	-0.10	-0.85	0.065
C. betulus	-0.10	-0.95	0.199
C. betulus	-0.10	-1.00	0.073
C. betulus	-0.125	-0.575	0.162
C. betulus	-0.13	-0.78	0.095
C. betulus	-0.18	-1.40	0.047
C. betulus	-0.20	-0.70	0.349
C. betulus	-0.20	-1.13	0.064
C. betulus	-0.25	-0.90	0.174
C. betulus	-0.25	-0.98	0.224
C. betulus	-0.25	-1.00	NA
C. betulus	-0.25	-1.00	0.056
C. betulus	-0.28	-0.80	0.087
C. betulus	-0.30	-0.70	NA
C. betulus	-0.30	-1.03	0.036
C. betulus	-0.30	-1.30	0.087
C. betulus	-0.33	-0.60	0.101
C. betulus	-0.33	-1.10	0.016
C. betulus	-0.35	-0.80	0.016
C. betulus	-0.38	-0.80	0.081
C. betulus	-0.38	-0.98	0.074
C. betulus	-0.38	-1.00	0.249
C. betulus	-0.38	-1.08	0.068
C. betulus	-0.38	-1.28	0.057
C. betulus	-0.38	-1.40	0.105
C. betulus	-0.40	-0.88	NA
C. betulus	-0.40	-1.00	0.099
C. betulus	-0.40	-1.03	0.059
C. betulus	-0.40	-1.05	0.140
C. betulus	-0.43	-0.85	0.092
C. betulus	-0.43	-1.13	0.025
C. betulus	-0.43	-1.43	0.031
C. betulus	-0.43	-1.75	0.136
C. betulus	-0.45	-0.90	NA
C. betulus	-0.45	-1.08	0.057

species	Ψpd (MPa)	Ψmd (MPa)	g_s (mol m^{-2} s^{-1})
C. betulus	-0.45	-1.10	0.061
C. betulus	-0.45	-1.13	0.101
C. betulus	-0.45	-1.30	0.058
C. betulus	-0.45	-1.43	0.186
C. betulus	-0.48	-1.23	0.198
C. betulus	-0.48	-1.30	0.030
C. betulus	-0.48	-1.50	0.094
C. betulus	-0.50	-0.75	0.260
C. betulus	-0.50	-0.80	0.035
C. betulus	-0.50	-1.08	0.062
C. betulus	-0.50	-1.23	0.073
C. betulus	-0.50	-1.33	0.030
C. betulus	-0.50	-1.48	0.018
C. betulus	-0.50	-1.50	0.051
C. betulus	-0.50	-1.55	0.043
C. betulus	-0.50	-1.65	0.053
C. betulus	-0.53	-0.70	0.130
C. betulus	-0.53	-1.33	0.025
C. betulus	-0.53	-1.53	0.043
C. betulus	-0.55	-0.95	0.233
C. betulus	-0.55	-1.13	0.029
C. betulus	-0.55	-1.15	0.027
C. betulus	-0.55	-1.28	0.120
C. betulus	-0.55	-1.35	0.033
C. betulus	-0.58	-1.23	0.023
C. betulus	-0.60	-1.30	0.053
C. betulus	-0.60	-1.33	0.043
C. betulus	-0.63	-1.20	0.059
C. betulus	-0.63	-1.35	0.047
C. betulus	-0.63	-1.40	0.055
C. betulus	-0.65	-1.30	0.085
C. betulus	-0.65	-0.95	0.046
C. betulus	-0.70	-1.40	0.070
C. betulus	-0.70	-1.43	0.251
C. betulus	-0.73	-1.48	-0.009
C. betulus	-0.73	-1.50	0.054
C. betulus	-0.75	-1.73	0.033
C. betulus	-0.78	-1.45	0.033
C. betulus	-0.78	-1.53	0.177
C. betulus	-0.78	-1.55	0.068
C. betulus	-0.85	-1.80	0.032
C. betulus	-0.90	-1.40	0.025
C. betulus	-0.90	-1.50	0.030
C. betulus	-0.93	-1.05	0.031
C. betulus	-0.95	-1.40	0.010
C. betulus	-0.98	-1.58	0.011
C. betulus	-1.23	-1.65	0.013
C. betulus	-1.25	-1.75	0.018
C. betulus	-1.28	-2.15	0.012
C. betulus	-1.40	-1.90	0.038
C. betulus	-1.65	-2.00	0.022
C. betulus	-1.70	-2.10	0.006
C. betulus	-1.78	-2.20	0.010
C. betulus	-1.85	-2.40	0.044
C. betulus	-1.95	-2.00	0.007
C. betulus	-2.05	-2.15	0.001
C. betulus	-2.10	-2.20	0.005
C. betulus	-2.10	-2.28	0.007
C. betulus	-2.65	-2.90	0.000
C. betulus	-3.08	-3.30	0.021
F. sylvatica	-0.20	-0.55	0.053
F. sylvatica	-0.20	-1.40	0.075
F. sylvatica	-0.28	-1.35	0.163
F. sylvatica	-0.30	-1.20	0.143
F. sylvatica	-0.30	-1.43	0.172
F. sylvatica	-0.33	-1.05	0.112
F. sylvatica	-0.33	-1.40	0.172
F. sylvatica	-0.35	-1.08	NA
F. sylvatica	-0.35	-1.43	0.080
F. sylvatica	-0.35	-1.60	0.215
F. sylvatica	-0.35	-1.65	0.050

species	Ψpd (MPa)	Ψmd (MPa)	g_s (mol m^{-2} s^{-1})
F. sylvatica	-0.40	-1.40	0.072
F. sylvatica	-0.43	-1.40	0.341
F. sylvatica	-0.43	-1.50	0.079
F. sylvatica	-0.43	-1.58	0.433
F. sylvatica	-0.43	-1.75	0.131
F. sylvatica	-0.45	-1.03	0.046
F. sylvatica	-0.45	-1.50	0.156
F. sylvatica	-0.45	-1.50	0.237
F. sylvatica	-0.45	-1.75	0.039
F. sylvatica	-0.48	-1.03	0.075
F. sylvatica	-0.48	-1.13	0.103
F. sylvatica	-0.48	-1.33	NA
F. sylvatica	-0.50	-1.48	0.057
F. sylvatica	-0.50	-1.53	0.142
F. sylvatica	-0.50	-1.58	0.104
F. sylvatica	-0.50	-1.63	0.098
F. sylvatica	-0.50	-1.75	0.134
F. sylvatica	-0.50	-1.95	0.097
F. sylvatica	-0.50	-2.00	0.233
F. sylvatica	-0.50	-2.15	0.163
F. sylvatica	-0.53	-0.95	0.034
F. sylvatica	-0.53	-1.30	0.068
F. sylvatica	-0.53	-1.60	0.070
F. sylvatica	-0.55	-0.95	0.035
F. sylvatica	-0.55	-1.15	0.102
F. sylvatica	-0.55	-1.35	NA
F. sylvatica	-0.55	-1.45	0.094
F. sylvatica	-0.55	-1.50	0.029
F. sylvatica	-0.55	-1.55	-0.003
F. sylvatica	-0.55	-1.88	0.160
F. sylvatica	-0.58	-1.25	NA
F. sylvatica	-0.58	-1.43	0.161
F. sylvatica	-0.58	-1.55	0.051
F. sylvatica	-0.58	-1.58	0.013
F. sylvatica	-0.58	-1.60	0.096
F. sylvatica	-0.58	-1.73	0.058
F. sylvatica	-0.58	-2.05	0.025
F. sylvatica	-0.60	-1.05	0.117
F. sylvatica	-0.60	-1.20	0.365
F. sylvatica	-0.60	-1.43	0.250
F. sylvatica	-0.60	-1.53	0.048
F. sylvatica	-0.63	-1.68	0.056
F. sylvatica	-0.63	-1.83	0.057
F. sylvatica	-0.65	-1.35	0.049
F. sylvatica	-0.65	-1.58	0.012
F. sylvatica	-0.65	-1.65	0.081
F. sylvatica	-0.65	-1.80	0.128
F. sylvatica	-0.65	-1.85	0.091
F. sylvatica	-0.65	-2.00	0.089
F. sylvatica	-0.65	-2.08	0.062
F. sylvatica	-0.68	-1.55	0.080
F. sylvatica	-0.68	-1.65	0.142
F. sylvatica	-0.68	-1.65	0.110
F. sylvatica	-0.68	-1.88	0.014
F. sylvatica	-0.68	-2.05	0.067
F. sylvatica	-0.70	-1.43	0.072
F. sylvatica	-0.70	-1.55	0.083
F. sylvatica	-0.70	-1.55	0.168
F. sylvatica	-0.70	-1.55	0.098
F. sylvatica	-0.70	-1.65	0.113
F. sylvatica	-0.70	-2.15	0.020
F. sylvatica	-0.73	-1.95	0.040
F. sylvatica	-0.73	-2.00	0.138
F. sylvatica	-0.73	-2.10	0.031
F. sylvatica	-0.75	-1.45	0.019
F. sylvatica	-0.75	-1.55	0.136
F. sylvatica	-0.75	-1.75	0.027
F. sylvatica	-0.75	-2.20	0.065
F. sylvatica	-0.78	-1.28	NA
F. sylvatica	-0.78	-1.35	0.038
F. sylvatica	-0.78	-1.43	0.103

species	Ψpd (MPa)	Ψmd (MPa)	g_s (mol m^{-2} s^{-1})
F. sylvatica	-0.78	-1.63	0.124
F. sylvatica	-0.78	-1.80	0.087
F. sylvatica	-0.78	-1.88	0.304
F. sylvatica	-0.80	-2.13	0.041
F. sylvatica	-0.83	-1.90	0.039
F. sylvatica	-0.83	-2.25	0.097
F. sylvatica	-0.85	-1.35	0.323
F. sylvatica	-0.85	-1.90	0.027
F. sylvatica	-0.85	-2.05	0.095
F. sylvatica	-0.88	-1.65	0.072
F. sylvatica	-0.88	-1.85	0.053
F. sylvatica	-0.90	-1.25	0.032
F. sylvatica	-0.93	-1.45	0.066
F. sylvatica	-0.93	-2.35	0.037
F. sylvatica	-0.95	-1.28	NA
F. sylvatica	-0.95	-1.98	0.041
F. sylvatica	-0.95	-2.05	0.037
F. sylvatica	-0.98	-1.50	0.045
F. sylvatica	-1.00	-1.90	0.097
F. sylvatica	-1.00	-1.98	0.064
F. sylvatica	-1.00	-2.03	0.092
F. sylvatica	-1.03	-2.05	0.035
F. sylvatica	-1.10	-2.45	0.016
F. sylvatica	-1.13	-2.15	0.024
F. sylvatica	-1.15	-2.35	0.015
F. sylvatica	-1.15	-2.35	0.051
F. sylvatica	-1.18	-2.00	0.076
F. sylvatica	-1.20	-2.50	0.048
F. sylvatica	-1.28	-2.73	0.038
F. sylvatica	-1.35	-2.13	0.027
F. sylvatica	-1.40	-2.03	0.066
F. sylvatica	-1.45	-2.23	0.022
F. sylvatica	-1.55	-2.65	0.030
F. sylvatica	-1.58	-2.75	0.050
F. sylvatica	-1.63	-2.18	0.079
F. sylvatica	-1.63	-2.55	0.025
F. sylvatica	-1.68	-2.60	0.026
F. sylvatica	-1.78	-2.30	0.028
F. sylvatica	-1.78	-2.50	0.034
F. sylvatica	-1.80	-2.60	0.031
F. sylvatica	-1.80	-2.70	0.010
F. sylvatica	-1.80	-2.73	0.008
F. sylvatica	-1.85	-2.30	0.080
F. sylvatica	-1.88	-2.40	0.007
F. sylvatica	-1.95	-2.43	0.003
F. sylvatica	-2.10	-2.80	0.004
F. sylvatica	-2.40	-2.90	0.015
F. sylvatica	-2.40	-3.05	0.014
F. sylvatica	-2.53	-2.80	-0.002
F. sylvatica	-2.63	-2.80	0.007
F. sylvatica	-2.75	-3.13	-0.001
F. sylvatica	-2.75	-3.15	0.018
F. sylvatica	-2.88	-3.23	0.007
F. sylvatica	-3.10	-3.50	0.007
F. sylvatica	-3.23	-3.80	0.016
F. excelsior	-0.23	-1.33	NA
F. excelsior	-0.28	-1.13	0.233
F. excelsior	-0.28	-1.13	NA
F. excelsior	-0.30	-1.60	0.277
F. excelsior	-0.33	-0.88	0.230
F. excelsior	-0.33	-1.23	NA
F. excelsior	-0.35	-1.60	0.189
F. excelsior	-0.38	-1.78	0.249
F. excelsior	-0.40	-0.60	0.070
F. excelsior	-0.40	-1.03	0.082
F. excelsior	-0.43	-0.80	0.273
F. excelsior	-0.48	-1.33	0.158
F. excelsior	-0.48	-1.38	0.154
F. excelsior	-0.48	-2.00	0.191
F. excelsior	-0.50	-0.58	0.106
F. excelsior	-0.60	-1.80	0.088

species	Ψpd (MPa)	Ψmd (MPa)	g_s (mol m^{-2} s^{-1})
F. excelsior	-0.63	-1.65	0.096
F. excelsior	-0.63	-1.80	0.030
F. excelsior	-0.63	-2.33	0.109
F. excelsior	-0.68	-1.63	0.047
F. excelsior	-0.68	-1.90	0.206
F. excelsior	-0.70	-1.48	-0.004
F. excelsior	-0.70	-2.13	0.257
F. excelsior	-0.70	-2.13	0.156
F. excelsior	-0.73	-1.43	0.059
F. excelsior	-0.73	-2.35	0.078
F. excelsior	-0.75	-1.43	0.061
F. excelsior	-0.75	-1.75	0.034
F. excelsior	-0.75	-2.15	0.102
F. excelsior	-0.75	-2.20	0.142
F. excelsior	-0.78	-1.65	0.056
F. excelsior	-0.80	-1.63	0.064
F. excelsior	-0.80	-2.05	0.093
F. excelsior	-0.83	-1.58	0.342
F. excelsior	-0.83	-2.00	0.142
F. excelsior	-0.83	-2.40	0.342
F. excelsior	-0.83	-2.43	0.103
F. excelsior	-0.83	-2.70	0.167
F. excelsior	-0.85	-2.35	0.050
F. excelsior	-0.85	-3.05	0.043
F. excelsior	-0.88	-1.90	0.234
F. excelsior	-0.90	-1.88	0.102
F. excelsior	-0.90	-2.00	0.104
F. excelsior	-0.90	-2.00	0.034
F. excelsior	-1.18	-2.85	0.040
F. excelsior	-1.43	-2.40	0.048
F. excelsior	-1.45	-2.60	0.060
F. excelsior	-1.75	-3.05	0.025
F. excelsior	-1.80	-3.00	0.047
F. excelsior	-2.00	-2.90	0.021
F. excelsior	-2.33	-3.40	0.024
F. excelsior	-2.50	-3.05	0.020
F. excelsior	-2.55	-2.80	0.006
F. excelsior	-2.58	-3.18	0.033
F. excelsior	-2.78	-3.35	0.021
P. abies	-0.50	-1.50	0.139
P. abies	-0.50	-1.63	0.123
P. abies	-0.53	-1.53	0.152
P. abies	-0.53	-1.65	0.186
P. abies	-0.53	-1.78	0.124
P. abies	-0.53	-1.80	0.102
P. abies	-0.55	-1.40	0.060
P. abies	-0.55	-1.83	0.113
P. abies	-0.58	-1.58	0.093
P. abies	-0.58	-1.65	0.086
P. abies	-0.60	-1.60	0.143
P. abies	-0.60	-1.75	0.152
P. abies	-0.60	-1.80	0.076
P. abies	-0.63	-1.78	0.061
P. abies	-0.65	-1.10	0.125
P. abies	-0.65	-1.45	0.019
P. abies	-0.65	-1.73	0.088
P. abies	-0.65	-1.85	0.128
P. abies	-0.65	-1.95	0.093
P. abies	-0.68	-1.33	0.040
P. abies	-0.68	-1.55	0.220
P. abies	-0.68	-1.70	0.035
P. abies	-0.68	-1.73	0.104
P. abies	-0.68	-2.00	0.036
P. abies	-0.68	-2.25	0.092
P. abies	-0.70	-1.23	0.138
P. abies	-0.70	-1.50	0.023
P. abies	-0.70	-1.60	0.076
P. abies	-0.70	-1.65	0.027
P. abies	-0.70	-1.70	0.071
P. abies	-0.70	-1.85	0.147
P. abies	-0.70	-1.98	0.063

species	Ψpd (MPa)	Ψmd (MPa)	g_s (mol m^{-2} s^{-1})
P. abies	-0.70	-2.00	0.119
P. abies	-0.70	-2.03	0.097
P. abies	-0.70	-2.33	0.103
P. abies	-0.73	-1.25	0.119
P. abies	-0.73	-1.40	NA
P. abies	-0.73	-1.63	NA
P. abies	-0.73	-1.70	0.102
P. abies	-0.73	-1.73	0.036
P. abies	-0.73	-1.85	0.148
P. abies	-0.73	-1.90	0.037
P. abies	-0.73	-1.98	0.126
P. abies	-0.73	-2.00	0.056
P. abies	-0.73	-2.08	0.080
P. abies	-0.73	-2.15	0.124
P. abies	-0.75	-1.33	0.105
P. abies	-0.75	-1.38	0.102
P. abies	-0.75	-1.53	0.049
P. abies	-0.75	-1.60	0.040
P. abies	-0.75	-1.65	0.070
P. abies	-0.75	-1.65	0.017
P. abies	-0.75	-1.73	0.061
P. abies	-0.75	-1.80	0.121
P. abies	-0.75	-1.80	0.059
P. abies	-0.75	-1.98	0.095
P. abies	-0.75	-1.98	0.125
P. abies	-0.75	-2.00	0.084
P. abies	-0.75	-2.03	0.162
P. abies	-0.75	-2.18	0.107
P. abies	-0.78	-1.40	0.079
P. abies	-0.78	-1.53	0.097
P. abies	-0.78	-1.65	0.057
P. abies	-0.78	-1.70	0.045
P. abies	-0.78	-1.78	0.099
P. abies	-0.78	-1.80	0.156
P. abies	-0.78	-1.80	0.095
P. abies	-0.78	-2.10	0.136
P. abies	-0.80	-1.30	0.100
P. abies	-0.80	-1.50	0.060
P. abies	-0.80	-1.65	0.013
P. abies	-0.80	-1.73	0.098
P. abies	-0.80	-1.78	0.097
P. abies	-0.80	-1.78	0.071
P. abies	-0.80	-1.83	0.126
P. abies	-0.80	-1.90	0.048
P. abies	-0.80	-1.90	0.040
P. abies	-0.83	-1.45	0.027
P. abies	-0.83	-1.63	0.005
P. abies	-0.83	-1.75	0.114
P. abies	-0.83	-1.75	0.030
P. abies	-0.83	-2.00	0.055
P. abies	-0.83	-2.18	0.043
P. abies	-0.85	-1.35	0.197
P. abies	-0.85	-1.43	0.118
P. abies	-0.85	-1.43	0.099
P. abies	-0.85	-1.50	0.046
P. abies	-0.85	-1.63	0.042
P. abies	-0.85	-1.75	0.038
P. abies	-0.85	-1.85	0.099
P. abies	-0.85	-1.85	0.133
P. abies	-0.85	-1.90	0.107
P. abies	-0.85	-2.23	0.072
P. abies	-0.85	-1.65	0.068
P. abies	-0.85	-1.65	0.046
P. abies	-0.88	-1.93	0.024
P. abies	-0.88	-2.10	0.041
P. abies	-0.90	-1.40	0.034
P. abies	-0.90	-1.95	0.075
P. abies	-0.90	-2.00	0.047
P. abies	-0.90	-2.03	0.083
P. abies	-0.90	-2.08	0.123
P. abies	-0.95	-1.65	0.072

species	Ψpd (MPa)	Ψmd (MPa)	g_s (mol m^{-2} s^{-1})
P. abies	-0.95	-2.15	0.039
P. abies	-0.98	-1.60	0.067
P. abies	-0.98	-1.60	0.157
P. abies	-0.98	-1.73	0.033
P. abies	-0.98	-2.10	0.037
P. abies	-1.00	-1.78	0.036
P. abies	-1.00	-1.85	0.101
P. abies	-1.03	-1.28	NA
P. abies	-1.03	-2.13	0.145
P. abies	-1.03	-2.13	0.089
P. abies	-1.03	-2.13	0.085
P. abies	-1.05	-1.90	0.052
P. abies	-1.05	-2.15	0.105
P. abies	-1.08	-1.63	0.029
P. abies	-1.10	-1.80	0.113
P. abies	-1.10	-2.00	0.087
P. abies	-1.10	-2.05	0.084
P. abies	-1.10	-2.10	0.112
P. abies	-1.20	-1.90	0.064
P. abies	-1.20	-2.08	0.030
P. abies	-1.20	-2.10	0.076
P. abies	-1.23	-1.68	0.024
P. abies	-1.23	-1.93	0.021
P. abies	-1.25	-1.75	0.060
P. abies	-1.25	-1.75	0.017
P. abies	-1.25	-1.85	0.039
P. abies	-1.28	-2.00	0.066
P. abies	-1.30	-2.00	0.084
P. abies	-1.33	-2.03	0.021
P. abies	-1.35	-1.90	0.105
P. abies	-1.35	-1.93	0.056
P. abies	-1.35	-2.15	0.024
P. abies	-1.45	-2.00	0.063
P. abies	-2.10	-2.20	0.004
P. abies	-2.10	-2.30	0.015
P. abies	-2.10	-2.30	0.009
P. abies	-2.10	-2.30	0.008
P. abies	-2.10	-2.35	0.002
P. abies	-2.18	-2.33	0.042
P. abies	-2.20	-2.50	0.007
P. abies	-2.25	-2.58	0.022
P. abies	-2.30	-2.40	0.044
P. abies	-2.30	-2.45	0.005
P. abies	-2.30	-2.55	0.024
P. abies	-2.35	-2.40	0.008
P. sylvestris	-0.40	-1.33	0.266
P. sylvestris	-0.55	-1.25	0.112
P. sylvestris	-0.55	-1.28	0.254
P. sylvestris	-0.55	-1.48	0.283
P. sylvestris	-0.58	-1.33	0.280
P. sylvestris	-0.60	-1.33	0.301
P. sylvestris	-0.60	-1.43	0.127
P. sylvestris	-0.60	-1.53	0.103
P. sylvestris	-0.60	-1.55	0.038
P. sylvestris	-0.63	-1.25	0.124
P. sylvestris	-0.63	-1.28	0.170
P. sylvestris	-0.63	-1.45	0.059
P. sylvestris	-0.63	-1.50	0.146
P. sylvestris	-0.63	-1.80	0.111
P. sylvestris	-0.65	-1.63	0.165
P. sylvestris	-0.65	-1.65	0.161
P. sylvestris	-0.65	-1.70	0.237
P. sylvestris	-0.68	-1.50	0.097
P. sylvestris	-0.68	-1.58	0.157
P. sylvestris	-0.68	-1.60	0.154
P. sylvestris	-0.70	-1.45	0.236
P. sylvestris	-0.70	-1.50	0.102
P. sylvestris	-0.70	-1.55	0.211
P. sylvestris	-0.70	-1.68	0.134
P. sylvestris	-0.70	-1.70	0.105
P. sylvestris	-0.70	-1.70	0.049

species	Ψpd (MPa)	Ψmd (MPa)	g_s (mol m^{-2} s^{-1})
P. sylvestris	-0.70	-1.80	0.204
P. sylvestris	-0.73	-1.28	0.052
P. sylvestris	-0.75	-1.70	0.090
P. sylvestris	-0.75	-1.83	0.110
P. sylvestris	-0.78	-1.25	0.172
P. sylvestris	-0.78	-1.35	-0.005
P. sylvestris	-0.78	-1.45	0.025
P. sylvestris	-0.80	-1.03	0.115
P. sylvestris	-0.80	-1.18	0.157
P. sylvestris	-0.80	-1.33	0.061
P. sylvestris	-0.80	-1.40	0.054
P. sylvestris	-0.80	-1.43	0.043
P. sylvestris	-0.80	-1.45	0.110
P. sylvestris	-0.80	-1.55	0.126
P. sylvestris	-0.80	-1.55	0.112
P. sylvestris	-0.80	-1.55	0.135
P. sylvestris	-0.80	-1.60	0.128
P. sylvestris	-0.80	-1.60	0.025
P. sylvestris	-0.80	-1.65	0.074
P. sylvestris	-0.80	-1.70	0.100
P. sylvestris	-0.80	-1.73	0.076
P. sylvestris	-0.83	-1.10	0.164
P. sylvestris	-0.83	-1.25	0.147
P. sylvestris	-0.83	-1.55	0.113
P. sylvestris	-0.85	-1.25	0.132
P. sylvestris	-0.85	-1.25	0.107
P. sylvestris	-0.85	-1.25	0.057
P. sylvestris	-0.85	-1.40	0.040
P. sylvestris	-0.85	-1.68	0.091
P. sylvestris	-0.88	-1.25	0.155
P. sylvestris	-0.88	-1.38	0.225
P. sylvestris	-0.88	-1.38	0.078
P. sylvestris	-0.88	-1.50	0.051
P. sylvestris	-0.88	-1.68	0.081
P. sylvestris	-0.88	-1.70	0.013
P. sylvestris	-0.88	-1.90	0.056
P. sylvestris	-0.90	-1.38	0.011
P. sylvestris	-0.90	-1.40	0.139
P. sylvestris	-0.90	-1.60	0.103
P. sylvestris	-0.90	-1.60	0.060
P. sylvestris	-0.90	-1.63	0.058
P. sylvestris	-0.90	-1.70	0.079
P. sylvestris	-0.90	-1.70	0.001
P. sylvestris	-0.93	-1.40	0.118
P. sylvestris	-0.93	-1.58	0.035
P. sylvestris	-0.93	-2.10	0.021
P. sylvestris	-0.95	-1.60	0.023
P. sylvestris	-0.95	-1.73	0.085
P. sylvestris	-0.98	-1.50	0.037
P. sylvestris	-0.98	-1.60	0.021
P. sylvestris	-0.98	-1.75	0.025
P. sylvestris	-1.00	-1.63	0.012
P. sylvestris	-1.03	-1.53	0.034
P. sylvestris	-1.03	-1.75	0.072
P. sylvestris	-1.05	-1.45	0.065
P. sylvestris	-1.05	-1.60	0.071
P. sylvestris	-1.05	-1.70	0.060
P. sylvestris	-1.05	-1.75	0.016
P. sylvestris	-1.13	-1.60	0.020
P. sylvestris	-1.20	-1.55	0.094
P. sylvestris	-1.20	-1.73	0.005
P. sylvestris	-1.20	-1.95	0.034
P. sylvestris	-1.25	-1.38	NA
P. sylvestris	-1.30	-1.80	0.027
P. sylvestris	-1.30	-1.83	0.004
P. sylvestris	-1.40	-1.90	0.018
P. sylvestris	-1.40	-2.10	0.051
P. sylvestris	-1.48	-1.88	0.017
P. sylvestris	-1.60	-1.73	0.016
P. sylvestris	-1.70	-1.83	0.004
P. sylvestris	-1.80	-1.88	0.003

species	Ψpd (MPa)	Ψmd (MPa)	g_s (mol m^{-2} s^{-1})
P. sylvestris	-1.80	-1.90	0.002
P. sylvestris	-1.80	-1.95	0.002
P. sylvestris	-1.80	-1.95	0.003
P. sylvestris	-1.80	-2.05	0.011
P. sylvestris	-1.83	-1.88	0.014
P. sylvestris	-1.85	-1.95	0.003
P. sylvestris	-1.93	-1.98	0.003
P. sylvestris	-2.00	-1.95	0.013
Q. spec.	-0.35	-1.10	0.322
Q. spec.	-0.35	-1.13	0.357
Q. spec.	-0.38	-1.35	0.367
Q. spec.	-0.38	-1.38	0.415
Q. spec.	-0.40	-1.03	0.361
Q. spec.	-0.43	-1.05	0.139
Q. spec.	-0.43	-1.18	0.091
Q. spec.	-0.45	-1.03	0.369
Q. spec.	-0.45	-1.33	NA
Q. spec.	-0.45	-1.58	0.248
Q. spec.	-0.45	-1.73	0.207
Q. spec.	-0.48	-1.00	0.125
Q. spec.	-0.48	-1.30	NA
Q. spec.	-0.50	-0.98	0.293
Q. spec.	-0.50	-1.13	NA
Q. spec.	-0.50	-1.13	NA
Q. spec.	-0.50	-1.20	0.189
Q. spec.	-0.50	-1.30	0.148
Q. spec.	-0.50	-1.90	0.091
Q. spec.	-0.53	-1.68	0.423
Q. spec.	-0.53	-1.80	0.337
Q. spec.	-0.55	-1.08	0.038
Q. spec.	-0.55	-1.10	0.148
Q. spec.	-0.55	-1.25	0.134
Q. spec.	-0.55	-1.38	0.072
Q. spec.	-0.55	-1.50	0.125
Q. spec.	-0.55	-1.55	0.503
Q. spec.	-0.55	-1.60	0.299
Q. spec.	-0.55	-2.10	0.179
Q. spec.	-0.58	-1.13	0.405
Q. spec.	-0.58	-1.55	0.314
Q. spec.	-0.58	-1.80	0.554
Q. spec.	-0.58	-1.98	0.265
Q. spec.	-0.60	-1.03	0.130
Q. spec.	-0.60	-1.05	0.169
Q. spec.	-0.60	-1.20	0.130
Q. spec.	-0.60	-1.20	0.048
Q. spec.	-0.60	-1.25	NA
Q. spec.	-0.60	-1.35	0.328
Q. spec.	-0.60	-1.60	0.327
Q. spec.	-0.60	-1.63	0.155
Q. spec.	-0.60	-1.70	0.399
Q. spec.	-0.60	-1.75	0.268
Q. spec.	-0.60	-1.85	0.372
Q. spec.	-0.60	-1.88	0.272
Q. spec.	-0.63	-1.05	0.189
Q. spec.	-0.63	-1.28	0.518
Q. spec.	-0.63	-1.40	0.179
Q. spec.	-0.63	-1.48	0.177
Q. spec.	-0.63	-1.83	0.180
Q. spec.	-0.63	-2.05	0.257
Q. spec.	-0.65	-1.58	0.173
Q. spec.	-0.65	-1.03	0.133
Q. spec.	-0.65	-1.08	0.173
Q. spec.	-0.65	-1.08	0.240
Q. spec.	-0.65	-1.08	0.132
Q. spec.	-0.65	-1.55	0.250
Q. spec.	-0.65	-1.65	0.182
Q. spec.	-0.65	-1.73	0.233
Q. spec.	-0.65	-1.78	0.277
Q. spec.	-0.65	-1.80	0.289
Q. spec.	-0.68	-1.23	0.354
Q. spec.	-0.68	-1.38	0.113

species	Ψpd (MPa)	Ψmd (MPa)	g_s (mol m^{-2} s^{-1})
Q. spec.	-0.68	-1.40	0.135
Q. spec.	-0.68	-1.43	0.106
Q. spec.	-0.68	-1.50	0.044
Q. spec.	-0.68	-1.58	0.067
Q. spec.	-0.68	-1.68	0.204
Q. spec.	-0.68	-1.70	0.054
Q. spec.	-0.68	-1.70	0.230
Q. spec.	-0.68	-1.83	0.245
Q. spec.	-0.68	-1.85	0.049
Q. spec.	-0.68	-2.15	0.437
Q. spec.	-0.70	-1.15	0.438
Q. spec.	-0.70	-1.55	0.096
Q. spec.	-0.70	-1.60	0.478
Q. spec.	-0.70	-1.63	0.053
Q. spec.	-0.73	-1.75	0.075
Q. spec.	-0.73	-1.80	0.344
Q. spec.	-0.75	-1.60	0.396
Q. spec.	-0.75	-2.10	0.031
Q. spec.	-0.78	-1.53	0.430
Q. spec.	-0.78	-1.65	0.437
Q. spec.	-0.78	-1.80	0.445
Q. spec.	-0.78	-2.00	0.133
Q. spec.	-0.80	-1.55	0.152
Q. spec.	-0.80	-1.65	0.296
Q. spec.	-0.80	-1.93	0.207
Q. spec.	-0.80	-2.00	0.102
Q. spec.	-0.80	-2.38	0.242
Q. spec.	-0.83	-1.58	0.445
Q. spec.	-0.83	-1.80	0.115
Q. spec.	-0.83	-1.95	0.132
Q. spec.	-0.85	-1.75	0.241
Q. spec.	-0.85	-1.98	0.082
Q. spec.	-0.85	-2.13	0.096
Q. spec.	-0.85	-2.58	0.271
Q. spec.	-0.88	-1.60	0.191
Q. spec.	-0.88	-1.85	0.104
Q. spec.	-0.88	-2.18	0.273
Q. spec.	-0.90	-1.78	0.124
Q. spec.	-0.90	-1.85	0.094
Q. spec.	-0.90	-2.23	0.148
Q. spec.	-0.90	-2.55	0.079
Q. spec.	-0.95	-1.95	0.112
Q. spec.	-0.95	-2.28	0.138
Q. spec.	-0.98	-2.15	0.069
Q. spec.	-0.98	-2.33	0.039
Q. spec.	-1.00	-2.20	0.112
Q. spec.	-1.00	-2.20	0.099
Q. spec.	-1.03	-2.20	0.072
Q. spec.	-1.13	-2.20	0.030
Q. spec.	-1.15	-2.85	0.032
Q. spec.	-1.20	-2.33	0.044
Q. spec.	-1.20	-2.60	0.113
Q. spec.	-1.30	-2.40	0.032
Q. spec.	-1.30	-2.50	0.042
Q. spec.	-1.30	-2.70	0.064
Q. spec.	-1.35	-2.45	0.064
Q. spec.	-1.45	-2.65	0.050
Q. spec.	-1.53	-2.90	0.038
Q. spec.	-1.55	-2.45	0.029
Q. spec.	-1.55	-2.70	0.083
Q. spec.	-1.58	-2.80	0.040
Q. spec.	-1.70	-2.75	0.063
Q. spec.	-1.75	-2.63	0.068
Q. spec	-1.85	-2.75	0.047
Q. spec	-1.85	-2.85	0.016
Q. spec	-1.85	-2.98	0.049
Q. spec	-1.90	-2.85	0.029
Q. spec	-1.90	-2.85	0.015
Q. spec	-2.10	-2.85	0.030
Q. spec	-2.13	-2.95	0.021
Q. spec	-2.28	-2.83	0.003
Q. spec	-2.45	-2.93	0.013

Table S2 Weather conditions in the observation years 2020 to 2022. Average temperature and sum of precipitation in the main vegetation period from May to August and deviation from long-term average (brackets). All data taken from the nearby climate station Rüneberg, operated by the Swiss Meteorological Service.

	**2020**	**2021**	**2022**
Average temperature May-Aug	17.2°C (+0.3 K)	15.6°C (-1.4 K)	19.1°C (+2.2 K)
Sum of precipitation May-Aug	369 mm (-11 %)	620 mm (+49 %)	289 mm (-30 %)

Table S3 Functional and ecological classification of the studied tree species compared to metrics of isohydrocity and stomatal regulation. The isohydrocity metrics slope and hydroscape area were derived from linear Ψ_{pd} *vs*. Ψ_{md} regressions. Critical predawn tree water potentials (Ψ_{pd}) for the onset of stomatal regulation (g_s) and metrics for stomatal sensitivity (ρ) were derived from asymptotic Ψ_{pd} *vs*. g_s regression models.

	P. abies	***A. alba***	***F. sylvatica***	***F. excelsior***	***C. betulus***	***A. pseudoplat.***	***Q. spec.***	***P. sylvestris***
functional type	coniferous	coniferous	diffuse-porous angiosperm	ring-porous angiosperm	diffuse-porous angiosperm	diffuse-porous angiosperm	ring-porous angiosperm	coniferous
drought tolerance[1]	1.8	1.8	2.4	2.5	2.7	2.8	3.0	4.3
slope Ψ_{pd} *vs.* Ψ_{md}	0.201	0.338	0.537	0.585	0.502	0.483	0.759	0.292
hydroscape area (MPa^2)	2.35	1.81	2.69	3.64	1.40	1.95	4.03	1.40
critical Ψ_{pd} for g_s regulation (MPa)	-0.81	-0.73	-0.61	-0.57	-0.42	-0.47	-0.63	-0.74
ρ, stomatal sensitivity	38	70	46	36	72	19	25	38

[1]Niinemets & Valladares, 2006

Table S4 Results from the subsampling analysis averaged across all subsampling proportions. Median (2.5 and 97.5 percentile) hydroscape area, mean (sd) best-fitting slope and adjusted R^2, percent of iterations which were discarded due to best-fitting slopes ≥ 1.

species	hydroscape area (95 perc.)	slope (sd)	R^2 (sd)	% slopes ≥ 1
P. sylvestris	1.21 (0.99, 1.87)	0.45 (0.14)	0.54 (0.16)	1.2
A. abies	1.55 (1.28, 1.99)	0.51 (0.17)	0.64 (0.16)	1.3
P. abies	2.08 (1.9, 2.29)	0.31 (0.08)	0.66 (0.16)	<0.1
A. pseudoplatanus	1.79 (1.28, 4.69)	0.75 (0.1)	0.82 (0.08)	11.1
C. betulus	1.3 (0.91, 5.32)	0.75 (0.1)	0.78 (0.1)	10.4
F. sylvatica	2.7 (1.95, 9.88)	0.76 (0.09)	0.8 (0.08)	9
F. excelsior	4.65 (2.52, 45.55)	0.84 (0.1)	0.74 (0.1)	30.9
Q. spec	6.7 (2.57, 138.74)	0.9 (0.13)	0.81 (0.07)	78.8

Notes S1 Random subsampling analysis of hydroscape areas (HAs). To estimate the reliability of the calculated HAs and the consistency of their ranking across species, we performed a subsample analysis with the full data (except the very few data points in the third phase of the dehydration trajectories in *F. sylvatica* and *C. Carpinus*). We randomly sampled different proportions of the data (90, 70, 50, 40, 30 and 20%) without replacement and calculated HAs from those subsamples as described in Material and Methods. Any iterations where the best-fitting slope was ≥ 1 were discarded, as the HA would effectively be infinite. From the remaining iterations, we found the median, 50% and 95% intervals of HAs. We did 1000 iterations per species and subsampling proportion, except for *F. excelsior* (1400) and *Q. spec*. (4800), as those two species had a high frequency of best-fitting slope being ≥ 1, which we had to discard (Tab. S4). The results of this subsample analysis showed low to moderate uncertainties in conifers and diffuse-porous angiosperms, respectively, but high uncertainty in the two ring-porous angiosperms *F. excelsior* and *Q. spec*. However, the ranking of the HAs across the species was robust (Fig. S1).

Reference

Niinemets Ü, Valladares F. 2006. Tolerance to shade, drought, and waterlogging of temperate Northern Hemisphere trees and shrubs. *Ecological Monogr*aphs **76**: 521-547.
